# Impact of Cross-Sectoral Video Consultation on Perceived Care Coordination and Information Satisfaction in Cancer Care: Randomized Controlled Trial

**DOI:** 10.2196/76910

**Published:** 2025-12-31

**Authors:** Fereshteh Baygi, Theis Bitz Trabjerg, Lars Henrik Jensen, Maria Munch Storsveen, Sonja Wehberg, Jens Søndergaard, Dorte Gilså Hansen

**Affiliations:** 1 Research Unit of General Practice, Department of Public Health, University of Southern Denmark Odense Denmark; 2 Lillebælt University Hospital, Department of Oncology Vejle Denmark; 3 Danish Colorectal Cancer Center South, Center of Clinical Excellence, Vejle Hospital, the Department of Regional Health Research, University of Southern Denmark Vejle Denmark

**Keywords:** randomized controlled trials, video consultations, outcome assessment, care coordination, information satisfaction, cancer

## Abstract

**Background:**

Enhancing care coordination and sharing information in cancer care improves patient experiences by promoting clarity and satisfaction.

**Objective:**

This study aims to assess the impact of cross-sectoral video consultation on patient perceptions of care coordination and satisfaction with received information compared to usual care.

**Methods:**

This study presents secondary outcomes on patient perceptions of care coordination and satisfaction with received information from a 7-month follow-up of the Partnership Project. In this randomized controlled trial, patients with cancer were allocated to either an intervention group receiving cross-sectoral video consultation (oncologist, general practitioner, and patient) or a control group receiving usual care. Patients’ perceptions of care coordination and information quality were assessed using the Australian Cancer Care Coordination Questionnaire (CCCQ) and the European Organisation for Research and Treatment of Cancer Quality of Life Information Questionnaire 25 at baseline and 7 months. Changes over time between groups were analyzed using generalized estimating equations.

**Results:**

Of the 278 participants randomized (1:1), only 80 (28.8%) patients received the intervention due to technical and administrative issues. A total of 210 (75.5%) patients completed the baseline questionnaire, while 118 (42.4%) responded at 7 months. No significant differences were observed in the changes over time between the intervention and control groups in any outcome. The estimated differences in the change in score from baseline to 7 months were as follows: for the total CCCQ score, 1.11 (95% CI –2.32 to 4.53; *P*=.53); for the overall European Organisation for Research and Treatment of Cancer Quality of Life Information Questionnaire 25 score, 1.49 (95% CI –2.98 to 5.96; *P*=.51); for the CCCQ communication subscale, –1.49 (95% CI –1.33 to 4.31; *P*=.30); and for the navigation subscale, –0.03 (95% CI –1.52 to 1.46; *P*=.97).

**Conclusions:**

Our findings indicate no statistically significant improvement in patients’ reported care coordination or satisfaction with received information over 7 months. Technical issues with the video setup reduced fidelity rates and follow-up participation. Further research is needed to optimize the structure and content of cross-sectoral video consultations to better support patients’ perceived outcomes.

**Trial Registration:**

ClinicalTrials.gov NCT02716168; https://clinicaltrials.gov/study/NCT02716168

## Introduction

The cancer care system is complex, involving numerous transitions between primary and specialist care, often characterized by inadequate communication among health care providers [1]. Recent literature has noted that the frequent interactions and involvement of multiple care providers in cancer treatment contribute to its complexity, making it particularly suitable for telehealth-based coordination approaches [2]. This complexity underscores the necessity of coordination for patients who are currently receiving or who have received cancer treatment, ensuring that the health care system delivers high-quality cancer care and improves patient experiences [3]. Effective collaboration between different sectors is crucial for maintaining continuity of services, avoiding unnecessary overlap, and ensuring that care remains focused on the needs of patients [4].

Several studies, both randomized and nonrandomized, have been conducted to improve care coordination and continuity of care for patients with cancer [4-8]. Despite progress in cancer care research, the findings remain controversial [4,6]. For example, a systematic review found that most studies reported no significant changes for patients (eg, quality of life); providers, including general practitioners’ (GPs) satisfaction with their role in patient care; or system outcomes (eg, frequency of GP visits). This review was unable to draw specific conclusions about the most effective models or interventions for improving cancer care coordination [6]. In contrast, a previous meta-analysis indicated that implementing cancer care coordination strategies led to improvements in most measured outcomes, such as overall patient experiences and quality of end-of-life care [4]. We believe this observed controversy highlights the challenges of developing effective interventions for the complex cancer care system, such as the mode of communication in care coordination and the degree of integration across sectors, which have not been addressed in previous studies [4].

None of the studies included in the previously mentioned systematic review [6] and meta-analyses [4] used a virtual intervention method to facilitate coordination. This gap is notable, particularly given the growing integration of digital health technologies into cancer care, including remote monitoring, patient portals, and electronic patient-reported outcomes, which have expanded significantly in recent years [9]. Among these, video consultation offers the potential to enable real-time, cross-sectoral dialogues [5]. Therefore, this study aimed to assess whether cross-sectoral video consultation can improve patient-perceived care coordination and information satisfaction over a 7-month period compared with usual care. We hypothesize that this approach will enhance these outcomes by facilitating timely, transparent, and inclusive communication across health care sectors, offering a potentially innovative solution to improve cancer care coordination.

## Methods

### Study Design

This study was a randomized controlled trial (RCT) titled the Partnership Project [10]. The protocol, details of this study, and findings on primary outcomes (eg, single-item global assessment of intersectoral cooperation) have been published previously [5,10,11]. This study presents secondary outcomes on patient perceptions of care coordination using the Australian Cancer Care Coordination Questionnaire (CCCQ) [12] and satisfaction with received information using the European Organisation for Research and Treatment of Cancer Quality of Life Information Questionnaire 25 (EORTC QLQ-INFO25) [13] from a 7-month follow-up survey in a shared video-based consultation.

### Participants and Setting

Patients diagnosed with any type of cancer and starting their first chemotherapy treatment at the Department of Oncology, Lillebælt Hospital, University Hospital of Southern Denmark, were considered newly diagnosed and invited to participate in this study. The eligibility criteria included (1) being aged >18 years, (2) being proficient in speaking and reading Danish, and (3) having an oncologist’s estimate of a survival time exceeding 7 months.

This trial was concluded upon reaching the predetermined sample size for patient inclusion.

### Sample Size

The sample size was based on estimates from a previous Danish RCT aiming to improve GP involvement in cancer follow-up. To detect a clinically meaningful difference with 90% power and a 5% significance level, 194 participants were required. Considering an anticipated dropout rate of 30%, the recruitment goal was set at 278 patients. Detailed information on sample size has been published previously [5,11].

### Intervention and Usual Care

Patients in the intervention group received a shared video consultation involving their GP, an oncologist, and themselves, in addition to usual care. These consultations were scheduled within 12 weeks of inclusion and could be held at either the GP’s or oncologist’s office based on patient preference. The oncologist typically chaired the session, supported by an oncology nurse, and both clinicians received a consultation guide beforehand.

The control group received usual care, which consisted of standard communication between the department of oncology and primary care, including electronic summary letters sent to the GP after each oncology visit, optional phone contact, and patient access to their GP.

A detailed description of the intervention and control groups, consultation structure, and implementation logistics has been published previously [5].

### Randomization and Blinding

Patients were assigned in a 1:1 ratio through block randomization, with block sizes and sequences managed by REDCap (Research Electronic Data Capture; Vanderbilt University) [14] data manager. A project nurse conducted the randomization and patient enrollment following informed consent. Allocation was transparent to patients, GPs, and oncologists, but blinding was maintained during baseline data collection. Data analysts remained blinded throughout. GPs of control group patients were not formally informed until they received the survey. Detailed information can be found in our previous publication [5].

### Outcomes and Instruments

Patients were asked to complete questionnaires at baseline and at 4 and 7 months. Upon arrival at the department of oncology, they received information about the study, their perceived role, and the possibility of leaving the study as well as a consent form and a paper-based baseline questionnaire, which was collected by an outpatient nurse after enrollment. Follow-up questionnaires were distributed electronically via REDCap [14]. An overview of the secondary outcomes in this study is provided in Multimedia Appendix 1. The outcomes included items and subscales from CCCQ [12] (22 items) and EORTC QLQ-INFO25 [13] (25 items).

For this study, the CCCQ was translated from English to Danish following the guidelines for cross-cultural adaptation of self-report measures proposed by Beaton et al [15]. The translation procedures were documented in separate reports using the template developed by the American Academy of Orthopaedic Surgeons and recommended by Beaton et al [15].

The CCCQ evaluates patients’ perceptions of cancer care coordination over a recall period of 3 months [12]. The CCCQ consists of 22 items: 2 global items, including global rating of coordination of care (item 21) and global rating of the quality of the received care (item 22) and 20 items that form 2 subscales, including the communication subscale (1-13; ranging from 13 to 65) and navigation subscale (items 14-20; ranging from 7 to 35). Responses are provided on 5-point Likert scales, ranging from “never” (1) to “always” (5), except for the global items, which use a 10-point Likert scale, ranging from “very poor” (1) to “very good” (10).

EORTC QLQ-INFO25, consisting of 25 items, assesses various aspects of patient satisfaction with the information provided during cancer treatment. Although no specific recall period was defined, patients were asked to consider their current cancer trajectory. The questionnaire includes a total score, 4 subscales covering information about the disease (items 1-4), medical tests (items 5-7), treatment (items 8-13) and other services (items 14-17) as well as 8 single items (items 18-25) addressing information about different care locations, self-help resources, written material, audiovisual formats, satisfaction with the information received, the need for more or less information, and the overall helpfulness of the information. In total, 4 additional yes-or-no questions explored the formats and quantity of information received by participants. Specifically, they asked whether participants had received written materials or audiovisual content (eg, CD, tape, or video), whether they wished to receive information (with an open-ended follow-up on preferred topics), and whether they felt they had received too much information (also with an option to specify topics). Responses to the core items were given on a 4-point Likert scale, ranging from “not at all” (1) to “very much” (4). All scores were linearly transformed to a scale ranging from 0 to 100 according to the scoring manual [13]. For the 4 items (written information, item 20; information on CD tape/video, item 21; wish to receive more information, item 23; and wish to receive less information, item 24) that have dichotomous response options (yes=1 or no=0), scores were treated as binary indicators.

Subscale score calculations were performed as follows:

- For CCCQ, subscale scores were also calculated as the mean of the items within each subscale. For Communication, up to 7 items could be missing, and for Navigation, up to 3 items could be missing. Scores of missing items were imputed using the mean of the nonmissing items within the respective subscale.
- For the EORTC QLQ-INFO25, subscale scores were calculated as the mean of the items within each subscale. For information about the disease, up to 2 single-item scores could be missing for the subscale score to remain valid. For information about medical tests, up to 1 item score could be missing; for information about treatments, up to 3 item scores could be missing; for information about other services, up to 2 item scores could be missing; for information about things you can do to help yourself, up to 13 scores could be missing. The remaining items were single items.

The direction of the answer scale varied by item. For the CCCQ Navigation subscale, a low score indicated a positive attitude toward the question, whereas for all other CCCQ subscales and single items, a high score indicated a positive attitude. For analysis purposes, all scores were aligned so that higher values indicated a positive attitude toward the questions. Specifically, item scores for items 14 to 20 were reversed coded to ensure consistent interpretation, such that an original score of 5 was recoded to 1, 4 to 2, 3 remained unchanged, 2 to 4, and 1 to 5.

Outcomes were measured at baseline and at 4 and 7 months after baseline. Coding was performed separately for each time point.

### Other Parameters

Demographic data for patients, including age, gender, education, marital status, having children, employment status, comorbidities, and cancer diagnosis or type, were collected via a questionnaire completed by patients at baseline.

### Statistical Analysis

A deviation from the initial analysis strategy was due to substantial missing data across all outcome variables at the 7-month follow-up (refer to the sample size reported for each outcome variable in the Results section). Detailed information on the original statistical analyses can be found in the published protocol [10] and our previous publication [5]. The revised statistical analysis is described subsequently.

For each secondary outcome, we compared the change from baseline to 7 months between the 2 groups using a linear regression model, following an intention-to-treat principle. The model, applied to measurements at both baseline and 7 months, used generalized estimating equations to account for within-patient clusters, with robust variance estimation. The group difference was modeled as a time-by-group interaction. No additional covariates were included in this analysis. We assumed that missing data were missing completely at random, meaning that the probability of missingness was unrelated to unmeasured factors after accounting for treatment group and baseline outcome. The generalized estimating equation approach ensured robustness in the presence of missing data. In addition, we calculated the individual changes from baseline to follow-up, restricted to complete cases, and presented Cohen *d* as an effect size measure and *P* values based on a 2-sample 2-tailed *t* test (Multimedia Appendix 2).

Data analyses were performed using Stata (version 18; StataCorp) [16], with a significance level set at 5% (*P*<.05).

### Ethical Considerations

#### Statement Regarding Human Participant Research Ethics Review

Ethics approval was obtained from the Regional Ethics Committee on Biomedical Research in Denmark (S-20142000-138) and the Danish Data Protection Agency (2014-41-3534).

#### Informed Consent

Informed consent was obtained from patients at the Department of Oncology, Vejle Hospital, Denmark. Consent covered participation in the RCT, video recordings, and patient-reported assessments. Consent forms were securely stored at the Clinical Research Unit, Vejle Hospital. As the unit of randomization was the patient, consent from GPs was not legally required. However, oral consent was obtained from GPs whose patients were allocated to the intervention group, and written information about the study was distributed to all GPs in the Region of Southern Denmark before recruitment. GPs who declined participation were excluded from this study.

#### Privacy and Confidentiality Protection

All video consultations were conducted using the Region of Southern Denmark’s secure videoconferencing infrastructure, which ensured encrypted communication and excluded any involvement of third-party data processing. The intervention guide provided to oncologists and GPs included instructions for managing potentially sensitive patient disclosure with care and professionalism.

#### Compensation

Patients and oncologists did not receive financial compensation. GPs were reimbursed through the region’s standard payment system for participating in video consultations and completing questionnaires, in line with cross-sector cooperation agreements.

Further details on the ethical framework and implementation are available in our previous publication [5].

## Results

### Recruitment and Baseline Data

**Figure 1:**
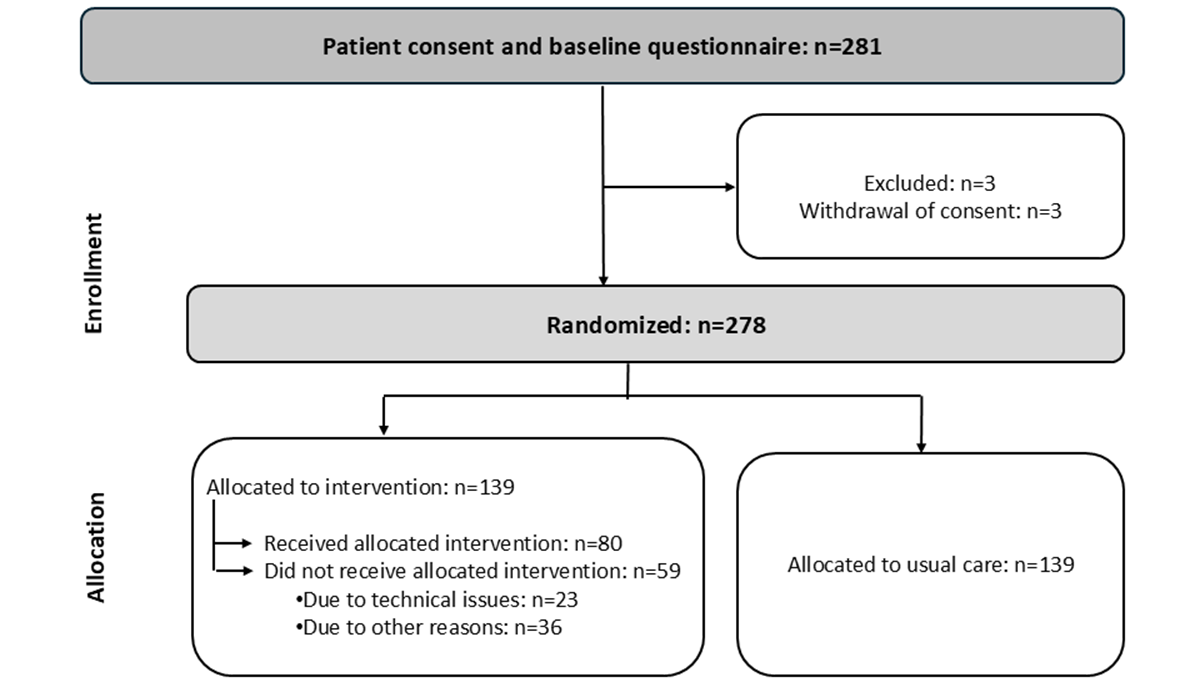
Flowchart of participant enrollment and allocation in a randomized controlled trial on cross-sectoral video consultation in cancer care in Denmark (between June 2016 and November 2019).

Patients were enrolled in this study between June 2016 and November 2019. A total of 278 patients were randomly assigned to the intervention (n=139, 50%) and control (n=139, 50%) groups. However, due to GP-related issues (n=22, 15.8%), administrative (n=8, 5.8%) and technical issues (n=15, 10.8%), and clinical (n=3, 2.2%) or patient-related issues (n=8, 5.8%), only 80 (57.6%) patients received the intervention as intended. Figure 1 shows the participation flowchart.

Table 1 presents the baseline characteristics of patients in the intervention and control groups. Patients in both groups exhibited similar baseline characteristics; however, comorbidities were more prevalent in the control group (n=81, 58.3%) compared to the intervention group (n=65, 46.8%).

**Table 1:** Baseline characteristics of patients in the intervention and control groups.

Characteristic			All (n=278)		Control group (n=139)		Intervention group (n=139)
Age (y), mean (SD)			65.2 (10.6)		63.8 (11.0)		66.6 (10.0)
**Sex, n (%)**							
	Male	155 (55.8)		77 (55.4)		78 (56.1)	
	Female	123 (44.2)		62 (44.6)		61 (43.9)	
**Education, n (%)**							
	Primary and upper secondary school	176 (63.3)		85 (61.2)		88 (63.3)	
	Further education (3-4 y)	76 (27.3)		41 (29.5)		35 (25.2)	
	Higher education (≥5 y)	16 (5.8)		7 (5.0)		9 (6.5)	
**Marital status, n (%)**							
	Single or information missing^a^	81 (29.1)		48 (34.5)		33 (23.7)	
	Married or residing with a companion	197 (70.9)		91 (65.5)		106 (76.3)	
**Children living at home, n (%)**							
	No children at home or information missing^a^	244 (87.8)		120 (86.3)		124 (89.2)	
	Children at home	34 (12.2)		19 (13.7)		15 (10.8)	
**Work status, n (%)**							
	Employed	89 (32)		46 (33.1)		43 (30.9)	
	Public benefits	15 (5.4)		9 (6.5)		6 (4.3)	
	Retired or information missing^a^	174 (62.6)		84 (60.4)		90 (64.7)	
**Comorbidity, n (%)**							
	No	132 (47.5)		58 (41.7)		74 (53.2)	
	Yes	146 (52.5)		81 (58.3)		65 (46.8)	
**Diagnosis or cancer type, n (%)**							
	Breast	33 (11.9)		17 (12.2)		16 (11.5)	
	Gynecologic	13 (4.7)		4 (2.9)		9 (6.5)	
	Lung	106 (38.1)		53 (38.1)		53 (38.1)	
	Gastrointestinal	110 (39.6)		56 (40.3)		54 (38.8)	
	Other	16 (5.8)		9 (6.5)		7 (5)	
	Incident cancer (yes or information missing^a^)	255 (91.7)		126 (90.6)		129 (92.8)	

^a^There were fewer than 3 patients with missing information on marital status, the number of children at home, or work status and 6 patients with missing information on cancer incidents. These patients were grouped with the indicated categories.

Multimedia Appendix 3 provides an overview of the missing data regarding patient care cooperation (CCCQ subscales) and information outcomes (EORTC QLQ-INFO25) at various time points for both the control and intervention groups. While the number and proportion of missing data vary across each variable, a consistent pattern was observed across all variables, where missing data were minimal at baseline but increased substantially at the 4- and 7-month follow-ups.

### Outcomes and Estimations

Table 2 presents patients’ perceptions of care coordination and satisfaction with the information received between the primary sector and the department of oncology. No statistically significant differences were observed in the changes over time between the intervention and control groups in any outcome.

**Table 2:** Patients’ perceptions of care coordination and satisfaction with the information received across the primary sector and the department of oncology in a randomized controlled trial in Denmark (between June 2016 and November 2019).^a^

Outcomes and group			Baseline		7 mo			Estimated change (95% CI)		Group-time interaction (95% CI)		*P* value
			n	Mean (SD)	n	Mean (SD)						
**Australian** **Cancer Care Coordination Questionnaire** **:** **subscores and single items**												
	**Global rating of coordination of care**											
		Control	134	8.37 (1.55)	59	8.59 (1.55)	0.14 (–0.23 to 0.51)		—^b^		—	
		Intervention	136	8.52 (1.40)	67	8.73 (1.67)	0.13 (–0.29 to 0.54)		–0.01 (–0.57 to 0.54)		.96	
	**Global rating of the quality of received care**											
		Control	136	8.74 (1.24)	59	9.08 (1.19)	0.21 (–0.06 to 0.48)		—		—	
		Intervention	135	8.86 (1.15)	67	8.91 (1.54)	–0.02 (–0.38 to 0.35)		–0.22 (–0.68 to 0.23)		.33	
	**Communications**											
		Control	135	46.79 (8.48)	59	47.80 (8.40)	0.16 (–2.11 to 1.80)		—		—	
		Intervention	134	48.13 (8.97)	68	50.06 (8.46)	–1.34 (0.70 to 3.37)		–1.49 (–1.33 to 4.31)		.30	
	**Navigation**											
		Control	135	30.30 (3.80)	59	30.36 (4.78)	–0.43 (–1.49 to 0.62)		—		—	
		Intervention	135	30.79 (3.83)	67	30.63 (4.94)	–0.47 (–1.52 to 0.59)		–0.03 (–1.52 to 1.46)		.97	
	**Total**											
		Control	135	77.08 (10.79)	59	78.16 (11.33)	–0.84 (–3.27 to 1.59)		—		—	
		Intervention	133	60.42 (8.08)	67	58.60 (8.50)	0.26 (–2.15 to 2.67)		1.11 (–2.32 to 4.53)		.53	
**European Organisation for Research and Treatment Quality of Life Information Questionnaire 25** **:** **subscores and single items**												
	**Information about the disease**											
		Control	135	59.75 (22.04)	59	65.77 (22.37)	4.09 (–1.82 to 10.00)		—		—	
		Intervention	134	58.89 (20.83)	68	67.03 (24.75)	5.96 (0.47 to 11.44)		1.87 (–6.19 to 9.93)		.65	
	**Information about medical tests**											
		Control	135	76.09 (22.31)	60	77.22 (23.89)	–1.38 (−7.09 to 4.32)		—		—	
		Intervention	133	81.29 (18.62)	68	80.23 (23.34)	–2.23 (–7.49 to 3.02)		–0.85 (–8.61 to 6.91)		.83	
	**Information about treatment**											
		Control	134	65.17 (21.44)	60	65.54 (21.20)	–2.62 (–7.77 to 2.52)		—		—	
		Intervention	133	67.54 (20.22)	68	69.22 (24.45)	0.39 (–5.34 to 6.13)		3.02 (–4.69 to 10.72)		.44	
	**Information about other services**											
		Control	132	40.70 (26.31)	60	46.67 (28.01)	2.96 (–2.90 to 8.82)		—		—	
		Intervention	133	42.88 (26.23)	68	54.62 (30.60)	9.24 (2.43 to 16.04)		6.27 (–2.71 to 15.25)		.17	
	**Information about different places of care**											
		Control	129	37.21 (36.24)	59	44.07 (33.01)	1.75 (–6.40 to 9.90)		—		—	
		Intervention	131	36.90 (36.81)	67	47.76 (35.87)	10.49 (1.09 to 19.90)		8.74 (–3.70 to 21.19)		.17	
	**Information about things you can do to help yourself**											
		Control	131	55.47 (32.45)	59	58.76 (31.16)	–0.49 (–7.04 to 6.07)		—		—	
		Intervention	128	64.58 (29.80)	66	59.60 (33.34)	–7.50 (–15.23 to 0.22)		–7.02 (–17.15 to 3.11)		.17	
	**Satisfaction with the information received**											
		Control	136	83.33 (23.31)	59	80.79 (24.14)	–4.25 (–10.25 to 1.75)		—		—	
		Intervention	136	90.20 (17.27)	68	82.84 (22.67)	–6.81 (–11.65 to –1.97)		–2.56 (–10.27 to 5.15)		.52	
	**Overall, the information has been helpful**											
		Control	134	84.33 (21.51)	60	78.33 (24.41)	–7.57 (–13.20 to –1.93)		—		—	
		Intervention	135	89.38 (15.59)	68	84.31 (19.51)	–5.38 (–9.85 to –0.90)		2.19 (–5.01 to 9.38)		.55	
	**Written information**											
		Control	135	91.11 (28.56)	60	88.33 (32.37)	–3.27 (–11.61 to 5.08)		—		—	
		Intervention	134	88.81 (31.65)	67	80.60 (39.84)	–8.69 (–19.53 to 2.15)		–5.42 (–19.10 to 8.26)		.44	
	**Information on CD, tape, or video**											
		Control	134	3.73 (19.02)	57	3.51 (18.56)	0.71 (–3.19 to 4.61)		—		—	
		Intervention	135	4.44 (20.68)	65	1.54 (12.40)	–2.29 (–5.11 to 0.52)		–3.01 (–7.82 to 1.80)		.22	
	**Wish to receive more information**											
		Control	133	32.33 (46.95)	59	16.95 (37.84)	–15.08 (–26.39 to –3.76)		—		—	
		Intervention	136	24.26 (43.03)	64	12.50 (33.33)	–12.13 (–22.46 to –1.79)		2.95 (–12.37 to 18.27)		.71	
	**Global score**											
		Control	122	52.30 (12.96)	53	51.13 (13.81)	–3.53 (–6.34 to –0.72)		—		—	
		Intervention	124	54.62 (10.63)	55	52.94 (14.90)	–2.04 (–5.52 to 1.44)		1.49 (–2.98 to 5.96)		.51	

^a^The efficacy of the intervention was evaluated in unadjusted generalized estimating equation population-averaged linear regression models following an intention-to-treat approach, where the effectiveness was estimated as a group-time interaction term. For the control group, this within-group change was the estimated regression coefficient for time (follow-up vs baseline), while for the intervention group, the within-group change was based on the estimated coefficient for time and the estimated group-time interaction. The group effect was modeled as the difference between the within-group changes over time, that is, the coefficient of the group-time interaction term.

^b^Not applicable

The estimated within-group changes between baseline and 7-month follow-up for the total CCCQ score were –0.84 (95% CI –3.27 to 1.59; *P*=.498) in the control group and 0.26 (95% CI –2.15 to 2.67; *P*=.83) in the intervention group. The between-group difference was estimated as 1.11 (95% CI –2.32 to 4.53; *P*=.53). The estimated within-group changes between baseline and 7-month follow-up for the overall INFO25 score were –3.53 (95% CI –6.34 to –0.72; *P*=.01) in the control group and –2.04 (95% CI –5.52 to 1.44; *P*=.25) in the intervention group. The between-group difference was estimated as 1.49 (95% CI –2.98 to 5.96; *P*=.52). The estimated difference in the change from baseline to 7 months for the CCCQ communication subscale was –1.49 (95% CI –1.33 to 4.31; *P*=.30) and for the navigation subscale was –0.03 (95% CI –1.52 to 1.46; *P*=.97).

## Discussion

### Principal Findings

This study found that the addition of a cross-sectoral video consultation to usual care did not lead to statistically significant improvements in patients’ perceptions of care coordination and satisfaction with the information received over 7 months. There were no significant differences in changes over time between the control and intervention groups for any of the secondary outcomes.

### Comparison to Prior Work

Telemedicine, including video consultation and phone calls, has emerged as a crucial tool for maintaining the continuity of medical care for patients with various medical conditions during the COVID-19 pandemic [17]. However, our study on the innovative health care model was conducted before the COVID-19 pandemic and the implementation of standard video setups. Since then, the technical aspects of video-based communication in health care have significantly improved, making it challenging to compare our findings with post–COVID-19 pandemic studies that use these advanced protocols.

Despite this advancement, recent research highlights that the impact of telemedicine on patient outcomes remains nuanced [18,19]. For instance, a meta-analysis on the efficacy of telemedicine for outpatients found that video consultation was feasible but did not significantly outperform face-to-face care in oncology in terms of patient satisfaction and attendance [18]. Authors in an editorial with a conceptual perspective lens argued that while remote consultation may enhance access, they risk compromising rational continuity, information richness, and shared decision-making [19]. This underscores the importance of context-sensitive consultation approaches, guided by clinical judgment and patient preference rather than policy mandates [19]. We believe that while these 2 studies focus on different dimensions, both highlight limitations of telemedicine and reinforce the need for thoughtful implementation.

A systematic review that analyzed 51 studies across multiple medical disciplines, including primary care, oncology, dermatology, and so on, revealed that telemedicine provided valuable support to traditional medicine during the COVID-19 pandemic [17]. The review highlighted a high level of patient satisfaction with telemedicine, particularly in areas such as convenience, continuity of care, communication, and efficiency [17]. In contrast, our study, which focused on patients’ perception of care coordination and satisfaction with the information received, found no significant improvement in these areas after the follow-up period. This discrepancy might be due to the different variables and methodologies used in each study as well as several limitations in our study, such as many missing values at follow-up, the lack of standard protocols for video consultation due to the timing of the trial, and other implementation challenges, including technical issues or scheduling difficulties, that were comprehensively explained in our previous publication [5].

The other study comparing the content and quality of various modes of consultation (eg, video, telephone, and face-to-face) found that face-to-face consultations enabled more information exchange between GPs and patients compared to video and telephone consultations [20]. The observed difference between our study and the aforementioned study highlights the potential limitations of video consultation in achieving patient outcomes and emphasizes the importance of face-to-face interaction in the health care setting. However, due to several limitations in our study that have been discussed in our previous publication [5], we believe that comparisons with other studies and the generalizability of our findings should be approached with caution. Specifically, the high rates of missing data in our study hinder us from drawing concrete conclusions about the intervention effect on patients’ perception of care coordination and satisfaction with the information received. Instead, we encourage the research community to learn from our trial’s challenges [5] and insights to improve future studies on this research area.

### Limitations of This Study

First, a key limitation of this study concerns the use of the CCCQ questionnaire, which was translated from English into Danish for this project. Although the translation followed the established guidelines for cross-cultural adaptation of self-report measures [15], the Danish version had not been validated before this study. This may impact the reliability and cultural appropriateness of the instrument in the Danish context. Second, the considerable amount of missing data observed at the 7-month follow-up reduced statistical power, affecting the ability to detect significant effects. Third, another limitation is the likely ceiling effect observed in both the CCCQ and EORTC QLQ-INFO25. At baseline, scores in both groups were high, leaving limited room for measurable improvement, potentially masking the intervention’s impact. Implementation challenges further limited this study. These included low completion rates of video consultations, technical and administrative issues, and the absence of a standardized video setup during the trial—details previously reported [5]. In addition, the absence of structured fidelity metrics, such as session counts, duration, and quality, limits the interpretability of intervention delivery. Although descriptive data on session uptake were collected, logistical challenges and a large amount of missing data prevented meaningful statistical analyses. Finally, the absence of statistically significant findings likely reflects a combination of factors, including limited intervention uptake, variability in participant engagement, measurement limitations (eg, the use of translated but unvalidated questionnaires), and inconsistent implementation. These findings highlight the need for future studies to use validated outcome measures and incorporate robust implementation and fidelity-tracking strategies.

### Implications and Future Directions

From the clinical and implementation perspective, the findings suggest that when baseline care is already perceived as high quality, additional interventions may offer limited incremental value. Future efforts to improve care coordination and patient information may be more appropriately targeted toward settings or populations where baseline performance is suboptimal, as there may be more potential for meaningful improvement in these contexts. In addition, future studies should incorporate structured fidelity metrics (eg, session completion rate) to better understand intervention delivery and identify implementation barriers. This would support more adaptation to clinical settings and enhance scalability within established frameworks, such as the Consolidated Framework for Implementation Research and the reach, effectiveness, adoption, implementation, and maintenance framework, which help guide and evaluate the adaptation, effectiveness, and sustainability of health interventions [21-23]. Furthermore, redesigning the intervention to address technical and administrative challenges, such as streamlining, scheduling, integrating automated reminders, and improving digital infrastructure, could enhance feasibility and uptake. Co-designing future iterations with end users may further improve usability and engagement, supporting more effective implementation and transferability to other settings.

## Appendix

Multimedia Appendix 1 Overview of secondary outcomes.

Multimedia Appendix 2 Individual differences for complete cases.

Multimedia Appendix 3 Overview of the missing data for patient’s care cooperation (CCCQ) and satisfaction of information received (EORTC QLQ-INFO25) as secondary outcomes in both control and intervention groups at baseline and follow-up.

Multimedia Appendix 4 CONSORT checklist.

## Abbreviations

CCCQ: Cancer Care Coordination Questionnaire
EORTC QLQ-INFO25: European Organisation for Research and Treatment Quality of Life Information Questionnaire 25
GP: general practitioner
RCT: randomized controlled trial
REDCap: Research Electronic Data Capture

## Data Availability

Data collection was conducted in compliance with Article 6 the General Data Protection Regulation, based on participants’ consent. This consent does not authorize public disclosure of the data; therefore, the dataset cannot be shared with unauthorized parties.
