# Supplementary material for: Impact of Cross-Sectoral Video Consultation on Perceived Care Coordination and Information Satisfaction in Cancer Care: Randomized Controlled Trial
Source: JMIR Form Res. 2025 Dec 31;9:e76910. doi: 10.2196/76910 (PMC12805320; doi:10.2196/76910)
Supplement: Multimedia Appendix 1 [file formative_v9i1e76910_app1.docx]

**Multimedia Appendix1:** Overview of secondary outcomes

| **Variable** | **Short name** | **Item number** | **Reversed**  **items** |
| --- | --- | --- | --- |
| **CCCQ** |  |  |  |
| GI1 | Global rating of co-ordination of care | n=1: 21 | - |
| GI2 | Global rating of the quality of the received care | n=1: 22 | - |
| comm  navi  total | Communication  Navigation  Total | n=13:1-13  n=7:14-20  n=20, 1-20 | -  14-20  14-20 |
| **EORTC QLQ-INFO25** |  |  |  |
| Info-dis | Information about the disease | n=4: 1-4 |  |
| Info-medt | Information about medical tests | n=3: 5-7 |  |
| Info-treat | Information about treatments | n=6: 8-13 |  |
| Info-thse | Information about other services | n=4: 14-17 |  |
| Info-difp | Information about different places of care | n=1: 18 |  |
| Info-help | Information about things you can do to help yourself | n=1:19 |  |
| Info-sat | Satisfaction with the information received | n=1:22 |  |
| Info-over | Overall, the information was helpful | n=1:25 |  |
| Info-wrin | Written information | n=1: 20 |  |
| Info-cd | Information on CD tape/video | n=1: 21 |  |
| Info-recmor | Wish to receive more information | n=1: 23 |  |
| Info-recmor | Wish to receive less information | n=1: 24 |  |
| Info-total |  | n=25: 1-25 |  |
