# Supplementary material for: Impact of Cross-Sectoral Video Consultation on Perceived Care Coordination and Information Satisfaction in Cancer Care: Randomized Controlled Trial
Source: JMIR Form Res. 2025 Dec 31;9:e76910. doi: 10.2196/76910 (PMC12805320; doi:10.2196/76910)
Supplement: Multimedia Appendix 2 [file formative_v9i1e76910_app2.docx]

**Multimedia Appendix2:** Individual differences for complete cases

| **Outcome⃰⃰** | **Control** | | **Intervention** | | **Group difference** | | |
| --- | --- | --- | --- | --- | --- | --- | --- |
|  | N | Mean (SD) | N | Mean (SD) | Mean (95% CI) | p-value | Cohen’s d |
| **CCCQ** |  |  |  |  |  |  |  |
| GI1 | 58 | -0.0(1.1) | 65 | 0.1(1.6) | -0.1(-0.6;0.3) | 0.567 | -0.104 |
| GI2 | 58 | 0.0(1.5) | 66 | 0.0(1.8) | -0.0(-0.6;0.6) | 0.921 | -0.018 |
| navi | 58 | 0.8(4.2) | 64 | 0.7(4.5) | 0.1(-1.5;1.6) | 0.927 | 0.017 |
| comm | 58 | 1.1(8.3) | 66 | -0.8(9.7) | 1.9(-1.3;5.1) | 0.248 | 0.209 |
| total | 58 | 1.9(10.1) | 64 | 0.5(10.8) | 1.4(-2.3;5.2) | 0.452 | 0.137 |
| **EORTC QLQ-INFO25** |  |  |  |  |  |  |  |
| Info-dis | 57 | -1.4(25.3) | 66 | -3.0(24.4) | 1.7(-7.2;10.6) | 0.711 | 0.067 |
| Info-medt | 58 | 4.5(23.8) | 66 | 3.7(22.8) | 0.8(-7.5;9.1) | 0.849 | 0.034 |
| Info-treat | 58 | 6.5(21.5) | 66 | 1.3(26.1) | 5.2(-3.3;13.8) | 0.230 | 0.217 |
| Info-thse | 58 | -0.7(24.8) | 66 | -7.4(30.1) | 6.7(-3.2;16.6) | 0.183 | 0.241 |
| Info-difp | 55 | 7.3(35.0) | 65 | -9.7(43.6) | 17.0(2.6;31.5) | 0.022 | 0.427 |
| Info-help | 55 | 3.6(27.0) | 62 | 9.7(33.8) | -6.0(-17.3;5.3) | 0.292 | -0.196 |
| Info-sat | 57 | 6.4(25.5) | 66 | 6.1(21.0) | 0.4(-7.9;8.7) | 0.930 | 0.016 |
| Info-over | 58 | 9.8(21.6) | 65 | 5.6(19.2) | 4.1(-3.2;11.4) | 0.264 | 0.203 |
| Info-wrin | 58 | 6.9(31.7) | 65 | 12.3(48.4) | -5.4(-20.2;9.4) | 0.471 | -0.131 |
| Info-cd | 55 | -1.8(13.5) | 63 | 1.6(12.6) | -3.4(-8.2;1.4) | 0.159 | -0.262 |
| Info-recmor | 57 | 14.0(51.5) | 62 | 14.5(50.7) | -0.5(-19.1;18.1) | 0.959 | -0.009 |
| Info-total | 48 | 5.0(10.3) | 50 | 2.3(13.4) | 2.8(-2.0;7.6) | 0.256 | 0.231 |
| Includes means (SD) within groups, mean group difference (control minus intervention), 95% confidence interval (CI), p-value from two-sample two-tailed t-test, and Cohen’s d as effect size measure.  ⃰For outcome details, see Multimedia Appendix 1. | | | | | | | |
