# Supplementary material for: Impact of Cross-Sectoral Video Consultation on Perceived Care Coordination and Information Satisfaction in Cancer Care: Randomized Controlled Trial
Source: JMIR Form Res. 2025 Dec 31;9:e76910. doi: 10.2196/76910 (PMC12805320; doi:10.2196/76910)
Supplement: Multimedia Appendix 3 [file formative_v9i1e76910_app3.docx]

**Multimedia Appendix3:** Overview of the missing data for patient’s care cooperation (CCCQ) and satisfaction of information received (EORTC QLQ-INFO25) as secondary outcomes in both control and intervention groups at baseline and follow-up

|  | | | Baseline | | 4 months | | 7 months | |
| --- | --- | --- | --- | --- | --- | --- | --- | --- |
| Outcome⃰⃰ | Group | Total  (n) | Data available  n (%) | Data missing  n (%) | Data available  n (%) | Data missing  n (%) | Data available  n (%) | Data missing  n (%) |
| CCCQ |  |  |  |  |  |  |  |  |
| Gl1 | All | 278 | 270 (97.1%) | 8 (2.9%) | 152 (54.7%) | 126 (45.3%) | 126 (45.3%) | 152 (54.7%) |
| GI1 | I | 139 | 136 (97.8%) | 3 (2.2%) | 84 (60.4%) | 55 (39.6%) | 67 (48.2%) | 72 (51.8%) |
| GI1 | C | 139 | 134 (96.4%) | 5 (3.6%) | 68 (48.9%) | 71 (51.1%) | 59 (42.4%) | 80 (57.6%) |
| GI2 | T | 278 | 271 (97.5%) | 7 (2.5%) | 152 (54.7%) | 126 (45.3%) | 126 (45.3%) | 152 (54.7%) |
| GI2 | I | 139 | 135 (97.1%) | 4 (2.9%) | 84 (60.4%) | 55 (39.6%) | 67 (48.2%) | 72 (51.8%) |
| GI2 | C | 139 | 136 (97.8%) | 3 (2.2%) | 68 (48.9%) | 71 (51.1%) | 59 (42.4%) | 80 (57.6%) |
| comm | T | 278 | 269 (96.8%) | 9 (3.2%) | 153 (55.0%) | 125 (45.0%) | 127 (45.7%) | 151 (54.3%) |
| comm | I | 139 | 134 (96.4%) | 5 (3.6%) | 84 (60.4%) | 55 (39.6%) | 68 (48.9%) | 71 (51.1%) |
| comm | C | 139 | 135 (97.1%) | 4 (2.9%) | 69 (49.6%) | 70 (50.4%) | 59 (42.4%) | 80 (57.6%) |
| navi | T | 278 | 270 (97.1%) | 8 (2.9%) | 152 (54.7%) | 126 (45.3%) | 126 (45.3%) | 152 (54.7%) |
| navi | I | 139 | 135 (97.1%) | 4 (2.9%) | 83 (59.7%) | 56 (40.3%) | 67 (48.2%) | 72 (51.8%) |
| navi | C | 139 | 135 (97.1%) | 4 (2.9%) | 69 (49.6%) | 70 (50.4%) | 59 (42.4%) | 80 (57.6%) |
| total | T | 278 | 268 (96.4%) | 10 (3.6%) | 152 (54.7%) | 126 (45.3%) | 126 (45.3%) | 152 (54.7%) |
| total | I | 139 | 133 (95.7%) | 6 (4.3%) | 83 (59.7%) | 56 (40.3%) | 67 (48.2%) | 72 (51.8%) |
| total | C | 139 | 135 (97.1%) | 4 (2.9%) | 69 (49.6%) | 70 (50.4%) | 59 (42.4%) | 80 (57.6%) |
| EORTC QLQ-INFO25 |  |  |  |  |  |  |  |  |
| dis | T | 278 | 269 (96.8%) | 9 (3.2%) | 152 (54.7%) | 126 (45.3%) | 127 (45.7%) | 151 (54.3%) |
| dis | I | 139 | 134 (96.4%) | 5 (3.6%) | 84 (60.4%) | 55 (39.6%) | 68 (48.9%) | 71 (51.1%) |
| dis | C | 139 | 135 (97.1%) | 4 (2.9%) | 68 (48.9%) | 71 (51.1%) | 59 (42.4%) | 80 (57.6%) |
| medt | T | 278 | 268 (96.4%) | 10 (3.6%) | 152 (54.7%) | 126 (45.3%) | 128 (46.0%) | 150 (54.0%) |
| medt | I | 139 | 133 (95.7%) | 6 (4.3%) | 84 (60.4%) | 55 (39.6%) | 68 (48.9%) | 71 (51.1%) |
| medt | C | 139 | 135 (97.1%) | 4 (2.9%) | 68 (48.9%) | 71 (51.1%) | 60 (43.2%) | 79 (56.8%) |
| treat | T | 278 | 267 (96.0%) | 11 (4.0%) | 152 (54.7%) | 126 (45.3%) | 128 (46.0%) | 150 (54.0%) |
| treat | I | 139 | 133 (95.7%) | 6 (4.3%) | 84 (60.4%) | 55 (39.6%) | 68 (48.9%) | 71 (51.1%) |
| treat | C | 139 | 134 (96.4%) | 5 (3.6%) | 68 (48.9%) | 71 (51.1%) | 60 (43.2%) | 79 (56.8%) |
| thse | T | 278 | 265 (95.3%) | 13 (4.7%) | 150 (54.0%) | 128 (46.0%) | 128 (46.0%) | 150 (54.0%) |
| thse | I | 139 | 133 (95.7%) | 6 (4.3%) | 82 (59.0%) | 57 (41.0%) | 68 (48.9%) | 71 (51.1%) |
| thse | C | 139 | 132 (95.0%) | 7 (5.0%) | 68 (48.9%) | 71 (51.1%) | 60 (43.2%) | 79 (56.8%) |
| difp | T | 278 | 260 (93.5%) | 18 (6.5%) | 146 (52.5%) | 132 (47.5%) | 126 (45.3%) | 152 (54.7%) |
| difp | I | 139 | 131 (94.2%) | 8 (5.8%) | 82 (59.0%) | 57 (41.0%) | 67 (48.2%) | 72 (51.8%) |
| difp | C | 139 | 129 (92.8%) | 10 (7.2%) | 64 (46.0%) | 75 (54.0%) | 59 (42.4%) | 80 (57.6%) |
| help | T | 278 | 259 (93.2%) | 19 (6.8%) | 152 (54.7%) | 126 (45.3%) | 125 (45.0%) | 153 (55.0%) |
| help_ | I | 139 | 128 (92.1%) | 11 (7.9%) | 84 (60.4%) | 55 (39.6%) | 66 (47.5%) | 73 (52.5%) |
| help | C | 139 | 131 (94.2%) | 8 (5.8%) | 68 (48.9%) | 71 (51.1%) | 59 (42.4%) | 80 (57.6%) |
| sat | T | 278 | 272 (97.8%) | 6 (2.2%) | 153 (55.0%) | 125 (45.0%) | 127 (45.7%) | 151 (54.3%) |
| sat | I | 139 | 136 (97.8%) | 3 (2.2%) | 84 (60.4%) | 55 (39.6%) | 68 (48.9%) | 71 (51.1%) |
| sat | C | 139 | 136 (97.8%) | 3 (2.2%) | 69 (49.6%) | 70 (50.4%) | 59 (42.4%) | 80 (57.6%) |
| over | T | 278 | 269 (96.8%) | 9 (3.2%) | 153 (55.0%) | 125 (45.0%) | 128 (46.0%) | 150 (54.0%) |
| over | I | 139 | 135 (97.1%) | 4 (2.9%) | 84 (60.4%) | 55 (39.6%) | 68 (48.9%) | 71 (51.1%) |
| over | C | 139 | 134 (96.4%) | 5 (3.6%) | 69 (49.6%) | 70 (50.4%) | 60 (43.2%) | 79 (56.8%) |
| wrin | T | 278 | 269 (96.8%) | 9 (3.2%) | 152 (54.7%) | 126 (45.3%) | 127 (45.7%) | 151 (54.3%) |
| wrin | I | 139 | 134 (96.4%) | 5 (3.6%) | 84 (60.4%) | 55 (39.6%) | 67 (48.2%) | 72 (51.8%) |
| wrin | C | 139 | 135 (97.1%) | 4 (2.9%) | 68 (48.9%) | 71 (51.1%) | 60 (43.2%) | 79 (56.8%) |
| cd | T | 278 | 269 (96.8%) | 9 (3.2%) | 151 (54.3%) | 127 (45.7%) | 122 (43.9%) | 156 (56.1%) |
| cd | I | 139 | 135 (97.1%) | 4 (2.9%) | 83 (59.7%) | 56 (40.3%) | 65 (46.8%) | 74 (53.2%) |
| cd | C | 139 | 134 (96.4%) | 5 (3.6%) | 68 (48.9%) | 71 (51.1%) | 57 (41.0%) | 82 (59.0%) |
| recmore | T | 278 | 269 (96.8%) | 9 (3.2%) | 152 (54.7%) | 126 (45.3%) | 123 (44.2%) | 155 (55.8%) |
| recmore | I | 139 | 136 (97.8%) | 3 (2.2%) | 83 (59.7%) | 56 (40.3%) | 64 (46.0%) | 75 (54.0%) |
| recmore | C | 139 | 133 (95.7%) | 6 (4.3%) | 69 (49.6%) | 70 (50.4%) | 59 (42.4%) | 80 (57.6%) |
| total | T | 278 | 246 (88.5%) | 32 (11.5%) | 138 (49.6%) | 140 (50.4%) | 108 (38.8%) | 170 (61.2%) |
| total | I | 139 | 124 (89.2%) | 15 (10.8%) | 76 (54.7%) | 63 (45.3%) | 55 (39.6%) | 84 (60.4%) |
| total | C | 139 | 122 (87.8%) | 17 (12.2%) | 62 (44.6%) | 77 (55.4%) | 53 (38.1%) | 86 (61.9%) |
| T: Total; C: Control; I: Intervention.  ⃰⃰ For outcome details, see Multimedia Appendix1. | | | | | | | | |
